# Hsa_circ_0013561 promotes progression of nasopharyngeal carcinoma by activating JAK2/STAT3 signaling pathway

**DOI:** 10.1016/j.bjorl.2023.101362

**Published:** 2023-11-20

**Authors:** Tian Kaisai, Zheng Mantang, Yuan Tailei, Zheng Liying, Chen Xiaoping, Jin Mingming, Zhang Yi

**Affiliations:** a Ningxia Medical University, Postgraduate Training Base in Shanghai Gongli Hospital, Shanghai, China; b Shanghai Pudong New Area Gongli Hospital, Department of Otorhinolaryngology & Head and Neck Surgery, Shanghai, China; c Shanghai University of Medicine and Health Sciences, Shanghai Key Laboratory of Molecular Imaging, Shanghai, China; d Affiliated Hospital of Hebei University, Baoding, China

**Keywords:** Nasopharyngeal carcinoma, Hsa_circ_0013561, JAK2/STAT3, EMT

## Abstract

**Objective:**

Nasopharyngeal Carcinoma (NPC) is a malignancy of epithelium of epithelium of the nasopharynx, with the highest incidence of otolaryngeal malignancies. A growing number of studies confirm that Circular RNA (circRNA) plays an important role in tumor development, including Hsa_circ_0013561. This study aims to elucidate the process and mechanism of NPC regulation hsa_circ_0013561.

**Methods:**

In this study, circRNA expression nodes and subcellular localization in NPC tissues were analyzed by fluorescence in situ hybridization. The expression of hsa_circ_0013561 in NPC cells was further clarified by RT-qPCR. At the same time, the lentivirus vector interfered by hsa_circ_0013561 was constructed and transfected. The cell proliferation was detected by CCK-8 method, EdU assay and plate cloning assay. The cell cycle and apoptosis were detected by flow cytometry, and the cell migration ability was detected by wound healing assay and Transwell assay. Western blotting examined the expression of apoptosis, Epithelial-Mesenchymal Transition (EMT)-associated proteins, and Janus Kinase/Signal Transductor and Activator of Transcription (JAK/STAT) signaling pathway-related proteins.

**Results:**

The results showed that the expression of hsa_circ_0013561 in NPC samples was significantly upregulated and hsa_circ_0013561 localized in the cytoplasm. After down-regulating hsa_circ_0013561 expression, it significantly inhibited the proliferation and metastasis ability of NPC, inhibited EMT progression, and promoted apoptosis. Further studies showed that interference hsa_circ_0013561 significantly inhibited JAK2/STAT3 signaling pathway activation and induced the expression of apoptosis-related proteins.

**Conclusion:**

In summary, we found that hsa_circ_0013561 is a pro-tumor circRNA in NPC, which can reduce the activation of JAK2/STAT3 pathway by knocking down hsa_circ_0013561, thereby slowing down the malignant progression of NPC.

**Oxford Centre for Evidence-Based Medicine 2011 Levels of Evidence:**

Level 4.

## Highlights

• Hsa_circ_0013561 is significantly up regulated in nasopharyngeal carcinoma.
• Knockdown of Hsa_circ_0013561 in NPC cells inhibited cell proliferation and migration.
• Knockdown of Hsa_circ_0013561 in NPC cells inhibited EMT progression in vitro.
• Hsa_circ_0013561 functions through the JAK2/STAT3 signaling pathway.

## Introduction

Nasopharyngeal Carcinoma (NPC) has ethnic susceptibility and regional concentration, and the incidence area is concentrated in South China, East China, and West China, which is a cancer with 'Chinese characteristics'. In 2020, there were about 133,354 newly diagnosed cases of NPC worldwide, and 80,008 new deaths in the same year. More than 70% of these cases occurred in endemic regions, and the incidence of men in Southeast Asia ranked first in the world.^1^, ^2^ With the exploration of various new treatment methods, the recurrence, metastasis rate and mortality of NPC are gradually decreasing, however, some patients still face the risk of recurrence or metastasis.^3^ Therefore, there is an urgent need to explore the mechanism of NPC and develop new therapeutic methods to improve the prognosis of NPC.

Janus Kinase (JAK)/Signal Transducer and Activator of Transcription (STAT) pathway plays a central role in immune response, cell proliferation and differentiation, and studies have shown that overexpression and overactivation of components in the JAK/STAT pathway are associated with the development of different types of cancer.^4^ In the public transcriptomic analysis of NPC, JAK2 is the most significant upregulated gene associated with JAK activity activation, and JAK2 protein expression also has an impact on the prognosis of patients with NPC.^5^ IL-6 is elevated in the serum of patients with NPC. As the upstream activator of the JAK2/STAT3 pathway, the increased expression of IL-6 and the activation of JAK2/STAT3 signaling pathway have an impact on the prognosis of patients with NPC, shortening the survival time and providing potential targets for the treatment of NPC.^6^ Therefore, we can further dissect whether Hsa_circ_0013561 promotes the malignant progression of NPC by activating the JAK2/STAT3 pathway.

Circular RNAs (circRNAs) are a special class of non-coding RNA molecules that are formed by a non-canonical splicing mechanism of reverse splicing, connecting the 3' end of the exon to the 5' end of the same or upstream exon, forming a closed loop.^7^ At present, domestic and foreign studies have confirmed that circRNA can participate in the occurrence and development of laryngeal squamous cell carcinoma,^8^ bladder cancer,^9^ breast cancer,^10^ etc. In Non-Small Cell Lung Cancer (NSCLC), highly expressed circ_ZNF124 promotes the occurrence and development of NSCLC by promoting the activation of JAK2/STAT3 signaling pathway.^11^ In Hepatocellular Carcinoma (HCC), circ_0072088 activates JAK2/STAT3 signaling pathway and enhances the proliferation, migration, and invasion ability of HCC cells.^12^

Here, we aim to explore the biological role of hsa_circ_0013561 in NPC. We speculate that hsa_circ_0013561 regulates the role of JAK2/STAT3 signaling pathway in the progression of NPC, and we will further dissect the mechanism.

## Methods

### Tissue samples

Paraffin samples of carcinoma and paracancer tissues were collected from 3 patients with NPC who were pathologically diagnosed and had complete clinical data during 2021‒2022 from the Department of Pathology of Shanghai Gongli Hospital, and the paraffin samples were used for immunohistochemical experiments. This research was approved by the Ethics Committee of Shanghai Gongli Hospital, and all patients signed the informed consent.

### Fluorescence in situ hybridization (FISH)

We tested hsa_circ_0013561 with the help of Geneseed Biotech (Guangzhou, China). We used Cy3-bound anti-digoxin and FitC-bound anti-biotin antibodies to explore the signal. We used 4,6-Diamino-2- Phenylindole (DAPI) to invert the nucleus. After that, we used a Leica microscope to get the images.

### Cell culture and cell transfection

We purchased Human Nasal mucosal Epithelial Cells (HNEpC) from Shanghai phytobiotechnology Company (Shanghai, China) and received a donation from the NPC cell line HNE1 from the Naval Medical University. HNEpC cells were then inoculated in DMEM/F-12 (Gibco, GrandIsland, NY, USA) medium and HNE1 cells in RPMI-1640 (Gibco, GrandIsland, NY, USA) medium with 10% Fetal Bovine Serum (FBS, BIOEXPLORER FBS collected in South America) and 1% streptomycin/penicillin antibiotics, and cultured at 37 °C and 5% CO_2_.

We constructed lentivirus-stabilized hsa_circ_0013561 knockdown cells known as sh-circ0013561-HNE1. After cell passage, RNA was extracted to verify transfection efficiency.

### Quantitative real-time polymerase chain reaction (qRT-PCR)

Total RNA was isolated by the Fastagen kit (RNAfast200), and the amount of total RNA was measured by using the NanoDrop device (Thermo Fisher Scientific, Waltham, Massachusetts, USA). Complementary DNA (cDNA) is synthesized using the cDNA synthesis kit (Takara, Ozu, Japan). Primers were synthesized by Sangon Biotech (Shanghai, China) using Primer software based on the sequence of each gene in circBase. The hsa_circ_0013561 and β-actin primers used in this study are as follows: hsa_circ_0013561 (Forward: 5ʹ-GAATGCTGTCCTGTCCTC-3ʹ, Reverse: 5ʹ-TGCCAATCATGGTCAGAG-3ʹ), β-actin (Forward: 5ʹ-GGCTGTGCTATCCCTGTACG-3ʹ, Reverse: 5ʹ-CTTGATCTTCATTGTGCTGGGTG-3ʹ). β-actin is an endogenous control, while the 2^-ΔΔCt^ method is used for estimation of relative gene expression. Real-time PCR was performed in the StepOnePlus RT-PCR system (Thermo Fisher Scientific) using SYBR Green PCR Master Mix (Yeasen, Shanghai, China). The thermal cycle conditions of PCR were: 0.5 min at 95℃, 10 s at 95 ℃, 30 s at 60 ℃, and 40 cycles.

### Cell counting kit-8 (CCK-8) assay

According to the manufacturer's protocols, relative cell viability was measured at 24 h, 48 h, and 72 h after transfection using CCK-8 (Yeasen, Shanghai, China). The cells were inoculated in 96-well plates with 4,000 cells per well and 3 repeat wells. After 10 μL CCK-8 solution was added to each well, the plates were incubated at 37 °C for 1 h away from light. After 0 h, 24 h, 48 h, and 72 h, Optical Density (OD_450nm_) was measured at 450 nm for each sample using a microplate reader.

### 5-Ethynyl-2′-deoxyuridine (EdU) assay

We evaluated cell proliferation using the EdU assay kit (US EVERBRIGHT, Suzhou, China). Normal Control Cells (HNE1-NC) and sh-circ0013561-HNE1 cells in the logarithmic growth phase were inoculated in 24-well plates, inoculated with 4 × 10^4^ per well, and cultured overnight. Add 10 μL of EdU-labeled medium to a 24-well plate and then incubate at 5% CO_2_ at 37 °C for 2 h. After treatment with 4% paraformaldehyde and 0.5% Triton X-100, EdU and DAPI staining solutions were added to the staining and observed under fluorescence microscopy. The EdU incorporation rate is calculated as the ratio of the total number of EdU-positive cells (red signal) to DAPI-positive cells (blue signal).

### Colony formation assays

The transfected cells were inoculated into six-well plates at a density of 1000 cells per well and cultured in RPMI-1640 medium containing 10% FBS for 7 days. Washed with Phosphate Buffered Saline (PBS) twice, fixed with 4% paraformaldehyde for 20 min, and dyed with 0.5% crystal violet for 20 min. Colonies consisting of at least 50 cells were counted and compared with NC.

### Apoptosis test

We assessed apoptosis using apoptosis assays (BD Pharmingen, FITC Annexin V Apoptosis Detection Kit I). First, collect 1 × 10^6^ cells and add 100 μL of 1× Binding Buffer and transfer them to Eppendorf (EP) tubes, add 5 μL of Annexin V-APC and 5 μL PI to each tube. After mixing, incubate for 15 min at room temperature in the dark, add 500 μL of 1× Binding Buffer to terminate the reaction, and then detect apoptosis with flow cytometry.

### Cell cycle test

Cell cycle was assessed using a cell cycle assay kit (Beyotime, Cell Cycle and Apoptosis Analysis Kit). First, collect 1 × 10^6^ cells and add 500 μL staining buffer, 25 μL propidium iodide staining solution (20×) and 10 μL RNase A (50×) and transfer them to EP tubes, mix well and incubate at room temperature protected from light for 30 min. Measure the cell cycle with flow cytometry.

### Wound healing assay

Control cells and transfected cells were inoculated into six-well plates at a density of 3.5 × 10^5^ cells/well and cultured in RPMI-1640 medium containing 10% FBS. When confluence was reached at 90%‒95%, create a “scratch” using a 1 mL pipette tip and the scratched cells were washed off with PBS. Subsequently, basic medium without FBS was added for culture. At 0 h, 24 h, 48 h and 72 h, the wound area was measured under the microscope, and the wound area was measured with Image J software, and the cell scratch healing rate was calculated = [(scratch area at 0 h − scratch area at a certain time point)/scratch area at 0 h] × 100%.

### Transwell assay

Control cells and transfected cells were suspended in 200 μL serum-free medium at a density of 2 × 10^4^ cells/well and inoculated in the upper chamber of Transwell plate with 8 μm well. In addition, 600 μL medium containing 15% FBS was placed in the lower chamber as chemotactic agent. After 1 day of culture, it was fixed with 4% paraformaldehyde, stained with 0.1% crystal violet, and counted with 3 random fields of view under the microscope.

### Western blot analysis

Total proteins were isolated from transfected cells using RIPA Lysis Buffer (Beyotime, RIPA Lysis Buffer Strong) lysate with a protease inhibitor (Beyotime, Phenylmethanesulfonyl fluoride, PMSF). Protein concentration was determined with a BCA protein kit (Yeasen, Shanghai, China). Electrophoretic protein samples (15 μg) in SDS-PAGE (10%) gel and transfer them to PVDF membranes. Next, block with skim milk powder (5%) for 1 h and then incubate the primary antibody overnight in a 4 °C shaker. The corresponding primary antibody: anti-BAX (1:5000, Proteintech, Wuhan, China), anti-BCL-2 (1:5000, Proteintech, Wuhan, China), anti-E-cadherin (1:5000, Proteintech, Wuhan, China), anti-N-cadherin (1:5000, Proteintech, Wuhan, China), anti-JAK2 (1:1000, Affinity Biosciences, Jiangsu, China), anti-p-JAK2 (1:1000, Affinity Biosciences, Jiangsu, China), anti-STAT3 (1:1000, Zenbio, Chengdu, China), anti- p-STAT3 (1:1000, Zenbio, Chengdu, China) and anti-β-actin (1:10000, Proteintech, Wuhan, China). β-actin was used as internal reference. On the second day, incubated with peroxidase labeled secondary antibodies on a shaker for 1 h, the corresponding second antibody: Goat Anti-Mouse IgG (1:5000, Biosharp, Anhui, China) and Goat Anti-Rabbit IgG (1:5000, Biodragon, Beijing, China). Then ECL Western Blotting kit (Vazyme, Nanjing, China) was used to analyze the chromogenic protein bands. Finally, the bands were analyzed using Image J software.

### Statistical analysis

Differences between the groups were evaluated using the unpaired *t*-test, and the data were expressed as averages. We considered with *p* <  0.05 considered statistically significant. All statistical analyses were performed using GraphPad Prism.

## Results

### Has_circ_0013561 expression in NPC tissues and cells

**Figure 1:**
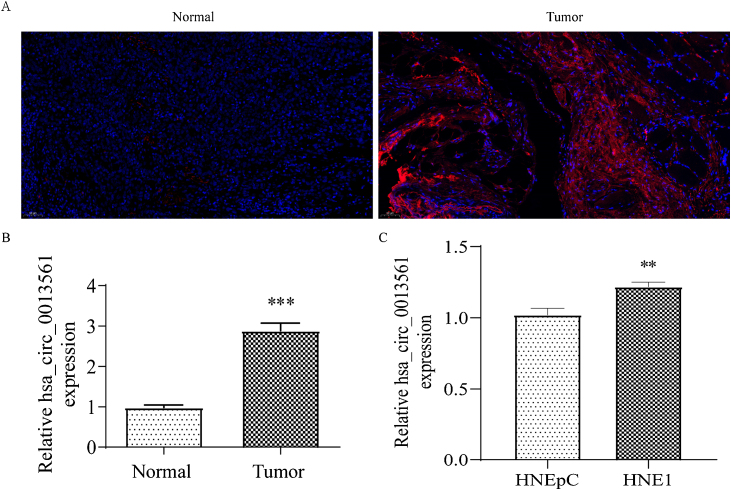
The expression of hsa_circ_0013561 were increased in both NPC tissues and cells. (A and B) FISH detection show the expression and subcellular localization of hsa_circ_0013561 in NPC tissues. Data are means ± SD; *** p < 0.001 vs normal. (C) RT-qPCR detection show the expression of hsa_circ_0013561 in HNE1 and HNEpC cells. Data are means ± SD; ** p < 0.01 vs HNEpC. Ruler scale = 50 μm.

To reveal the correlation between circRNA expression and nasopharyngeal carcinoma progression, we performed in situ hybridization on paraffin specimens from 3 nasopharyngeal carcinoma patients and analyzed nasopharyngeal carcinoma cells by qRT-PCR. FISH results showed that the hsa_circ_0013561 was significantly upregulated in cancerous tissue compared to paracancerous tissue and most of the hsa_circ_0013561 was distributed in the cytoplasm (Fig. 1A and B). Compared with HNEpC, hsa_circ_0013561 in NPC cells HNE1 was significantly up regulated (**p <  0.05, Fig. 1C).

### Knockdown hsa_circ_0013561 validated in NPC cells

**Figure 2:**
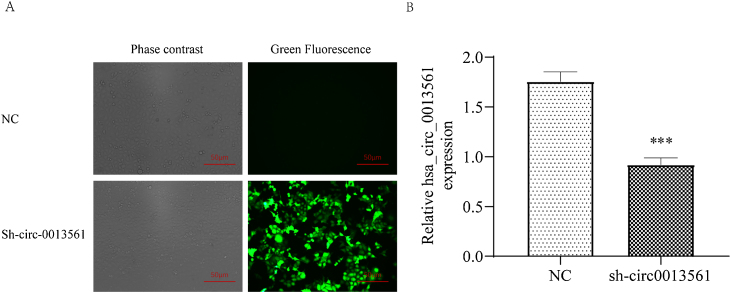
Knockdown of hsa_circ_0013561 was validated in cells. (A) Fluorescence observation of sh_circ_0013561cells transfected with lentivirus. (B) The expression of hsa_circ_0013561 in HNE1 cells was verified by qRT-PCR. Data are presented as mean ± SD; ** p < 0.05 vs. NC. Ruler scale = 50 μm.

Nasopharyngeal carcinoma cell line HNE1 was established, hsa_circ_0013561 knockdown virus model was constructed, and hsa_circ_0013561 was stably transfected into HNE1. After transfection, observe under a fluorescence microscope and take pictures (Fig. 2A). The results showed that the HNE1 cells in the knockdown group (sh-circ0013561) showed green fluorescence, while the Normal Control (NC) did not have green fluorescence. qRT-PCR results showed that compared with the NC group, the expression level of has_circ_0013561 in knockdown group was significantly down-regulated (***p <  0.05, Fig. 2B). The results indicated that the stable transfer sh-circ0013561 cell line was successfully constructed.

### Knockdown hsa_circ_0013561 inhibit cell proliferation

**Figure 3:**
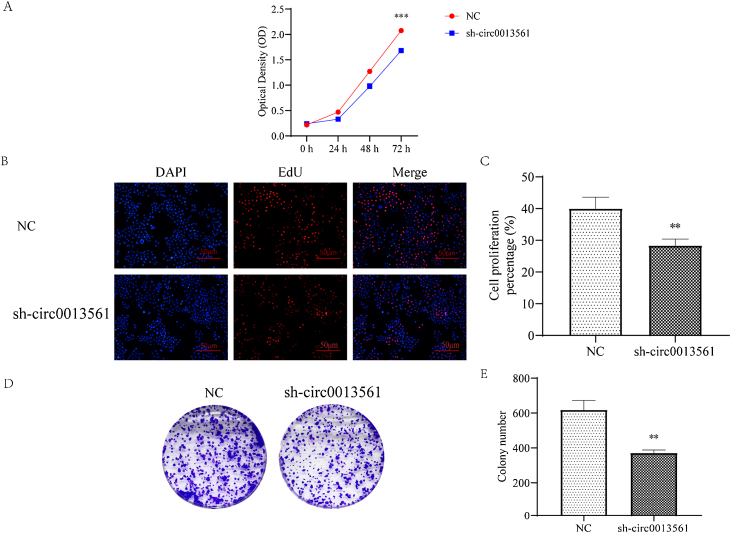
Knockdown of hsa_circ_0013561 inhibits NPC proliferation. (A) CCK8 assays were used to evaluate cell proliferation. Data are presented as mean ± SD; *** p < 0.001 vs. NC. (B and C) EdU assays were used to evaluate cell proliferation. Data are presented as mean ± SD; ** p < 0.05 vs. NC. (D and E) Colony formation assay showing proliferation in cells. Data are presented as mean ± SD; ** p < 0.05 vs. NC. Ruler scale = 100 μm.

CCK-8 (Fig. 3A), EdU tests (Fig. 3B and C) and plate cloning tests (Fig. 3D and E) showed that knockdown of hsa_circ_0013561 inhibited the proliferation of HNE1 cells. After 6 h of CCK8, cells were attached to the wall and OD value was measured at 0 h, and then measured for 3 consecutive days. The results showed that the mean OD value of 72 h in the knockdown group was significantly decreased compared with the mean OD value of 72 h in the NC group (***p <  0.05). In the EdU test, the count EdU/DAPI ratio was the percentage of cell proliferation, which was significantly decreased in knockdown group compared with the mean percentage of cell proliferation in NC group (**p <  0.05). Compared with the NC group, the average number of clone colonies in knockdown group was significantly decreased (**p < 0.05).

### Knockdown hsa_circ_0013561 alters apoptosis and cycle distribution

**Figure 4:**
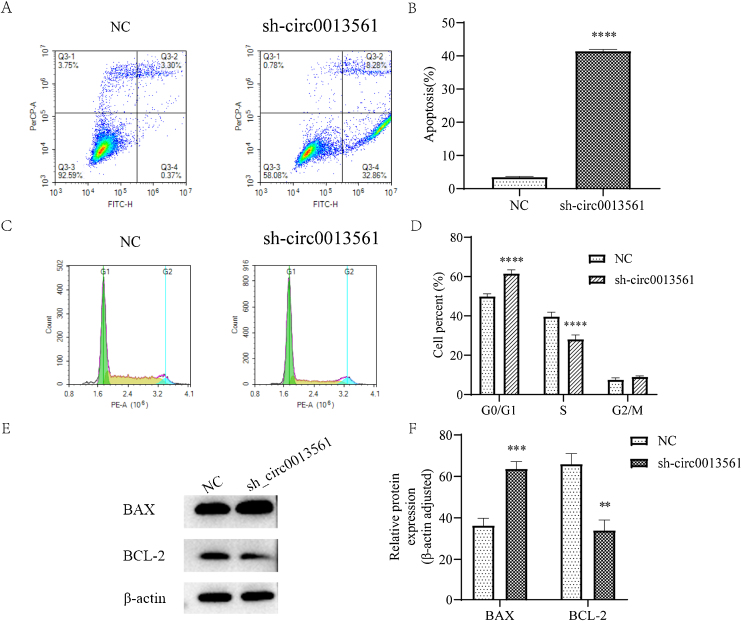
Knockdown of hsa_circ_0013561 changed cell apoptosis and cycle distribution. (A and B) The cell apoptosis was detected by flow cytometry. Data are presented as mean ± SD; **** p < 0.001 vs. NC. (C and D) The cell cycle was detected by flow cytometry, and the cell arrest was in G1 phase. Data are presented as mean ± SD; **** p < 0.001 vs.NC. (E-F) Relative expression of BAX and BCL-2 in HNE1 cells was detected by Western blot. The expression of BAX was increased and the expression of BCL-2 was decreased. Data are presented as mean ± SD; ** p < 0.05, *** p < 0.001 vs. NC.

Flow cytometry was used to detect apoptosis in the two groups (Fig. 4A and B), and the results showed that compared with the average apoptosis ratio of the NC group, the knockdown group was significantly increased (****p < 0.05), and the apoptosis ability of cells was enhanced after transfection knockdown hsa_circ_0013561. It has been shown that knocking down hsa_circ_0013561 promotes apoptosis in HNE1 cells. Cell cycle (Fig. 4C and D) HNE1 cells were stained with Propyl Iodide (PI), and cell cycle was detected by flow cytometry in both groups. The results showed that compared with the NC group, the average cell ratio of G0/G1 phase was significantly higher in the knockedown group (****p <  0.05). The results showed that compared with the NC group, the mean S phase cell ratio was significantly decreased in the knock down group (****p <  0.05). The results indicated that knockdown of hsa_circ_0013561 mediated the arrest of HNE1 cells in G1 phase. Western-blot assay was used to detect the expression of apoptosis-related proteins in the two groups (Fig. 4E‒F). Compared with the NC group, the knockdown group showed higher expression of apoptosis-related protein BAX and lower expression of anti-apoptosis-related protein BCL-2. The results indicated that hsa_circ_0013561 knockdown enhanced the expression of BAX protein in HNE1 cells, while decreased the expression of BCL-2 protein.

### Knockdown hsa_circ_0013561 inhibit cell migration and EMT progression

**Figure 5:**
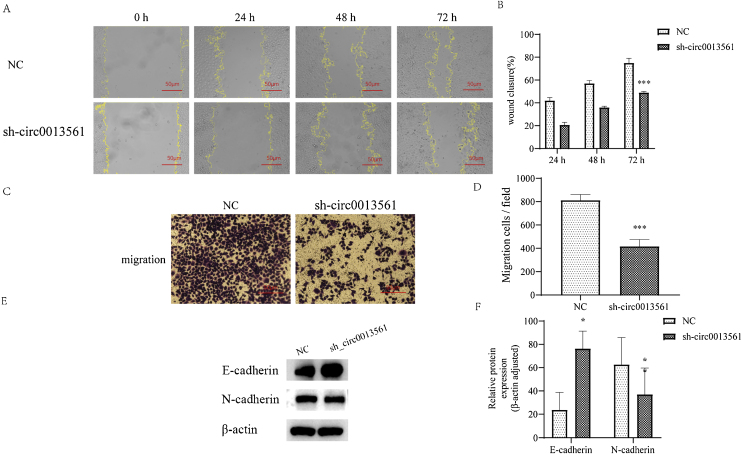
Knockdown of hsa_circ_0013561 inhibits NPC migration and EMT progression. (A and B) Cell migration ability was detected by wound healing assay. Data are presented as mean ± SD; *** p < 0.001 vs. NC. (C and D) Transwell assay was used to detect cell migration ability. Data are presented as mean ± SD; *** p < 0.001 vs.NC. (E and F) Relative expression of E-cadherin and N-cadherin in HNE1 cells was detected by Western blot. The expression of E-cadherin was increased and the expression of N-cadherin was decreased. Data are presented as mean ± SD; * p < 0.05, ** p < 0.05 vs. NC. Ruler scale = 50 μm.

Analysis of wound healing (Fig. 5A and B), which was used to measure migration ability, showed that the wound healing rate in the knock down group was significantly lower than the mean wound healing rate in the NC group at 72 h (***p <  0.05). In the results of transwell (Fig. 5C and D), the number of cells migrated per visual field was counted. Compared with the mean number of cells migrated in the NC group, the number of cells migrated in the knockdown group was significantly reduced (***p <  0.05). The expression of EMT-related protein in the two groups of cells was detected by the Western-blot method (Fig. 5E and F), and compared with the NC group, the knockdown group had high expression of the inhibition migration protein E-cadherin and low expression of the pro-migration protein N-cadherin. These results indicated that knockdown of hsa_circ_0013561 inhibited EMT progression in HNE1 cells.

### Knockdown hsa_circ_0013561 inhibit JAK2/STAT3 signaling pathway activation in NPC cells

**Figure 6:**
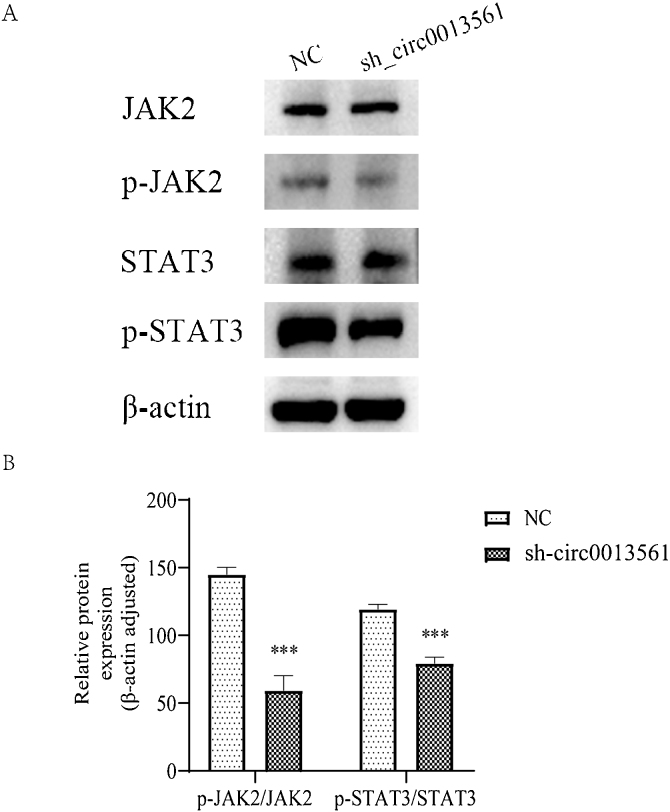
Knockdown hsa_circ_0013561 inhibit JAK2/STAT3 signaling pathway activation in NPC cells. (A) The expression of JAK2, p-JAK2, STAT3, and p-STAT3 proteins in cells after knocking down hsa_circ_0013561 in HNE1 cells. (B) The expression of p-JAK2 and p-STAT3 proteins was significantly downregulated, and the ratio of p-JAK2/JAK2 and p-STAT3/STAT3 decreased. Data are presented as mean ± SD; *** p < 0.05 vs. NC.

The Western-blot method was used to detect the expression of pathway-related proteins in the two groups (Fig. 6A), and compared with the NC group, the expression of p-JAK2 and p-STAT3 proteins was significantly downregulated, and the ratio of p-JAK2/JAK2 and p-STAT3/STAT3 decreased (Fig. 6B). These results indicated that hsa_circ_0013561 knockdown could inhibit the malignant progression of NPC by inhibiting the expression of JAK2/STAT3 signaling pathway related proteins.

## Discussion

The location of the nasal cavity is hidden, which makes it difficult to detect early NPC by general examination methods. So, most patients are already in the middle and late stage when detected, and some patients may have local and regional tumor recurrence or residual after the initial radical treatment, and the effects of radiotherapy and chemotherapy are not ideal.^13^, ^14^ Therefore, NPC has the characteristics of high incidence, difficult diagnosis and difficult surgical radical treatment, which makes it a difficult problem for surgeons, and it is particularly important to find new breakthroughs in treatment. circRNA contains a class of emerging rich endogenous noncoding RNA molecules, which play an important role in the pathogenesis and progression of cancer and has received extensive attention in recent years.^15^, ^16^ Studies have shown that circRNA has tissue and disease-specific expressibility and can be expressed differently in disease-associated genes.^16^, ^17^ Currently, many studies have shown that circRNA is involved in the occurrence and development of NPC, such as knockdown hsa_circ_0007637 inhibiting the growth of NPC cells, and hsa_circ_0007637 promoting the progression of NPC through the sponge miR-636/TPD52 axis.^18^ Circ_0000523 overexpression promoted the proliferation of NPC cells and accelerated the cell cycle process, while circ_0000523 knockdown inhibited the proliferation of NPC cells and induced cell cycle arrest.^19^

The best-known apoptosis-related family BCL-2 contains many pro-apoptotic proteins and anti-apoptotic proteins. Pro-apoptotic members such as BAX promote apoptosis and are the most important pro-apoptotic members of the BCL-2-related gene encoding the BCL-2 family, while anti-apoptotic members such as BCL-2 hinder the mechanism of apoptotic cell death.^20^, ^21^, ^22^ Liu et al.^23^ found that Ovatodiolide (Ova) inhibited tumor development, up-regulated the expression of BAX and down-regulated the expression of BCL-2, and increased the expression of E-cadherin. Ova inhibited the stem-like phenotype of NPC cells by inhibiting STAT3 signaling and regulating the dysfunction of JAK2/STAT3 pathway.

Epithelial-Mesenchymal Transition (EMT) plays an important role in cancer metastasis. EMT procedures can enhance metastasis, chemical resistance, and tumor stemness, mainly characterized by reduced expression of cell adhesion molecules such as E-cadherin. E-cadherin is a potential marker of malignancy.^24^, ^25^ N-cadherin is a transmembrane adhesion molecule associated with advanced cancer progression and poor prognosis. Increased expression of N-cadherin is a marker of EMT and promotes tumor cell survival, migration, and invasion.^26^, ^27^ Decreased or absent expression of E-cadherin can lead to weakened adhesion between cancer cells, which in turn makes cancer cells prone to shedding and metastasis, and promotes tumorigenesis, invasion and metastasis of epithelial tumors (including NPC).^28^, ^29^ Knockdown of the long non-coding RNA MALAT1 (Metastasis-Associated Lung Adenocarcinoma Transcription-1) inhibits proliferation, invasion, and EMT of NPC cells, as shown by increased expression of E-cadherin and decreased expression of N-cadherin.^30^

In this study, we found that hsa_circ_0013561 was significantly upregulated in NPC tissues and hsa_circ_0013561 was highly expressed in NPC cells HNE1 compared to HNEpC. These findings suggest that hsa_circ_0013561 may play a role in NPC progression. In order to further explore the function, expression and mechanism of hsa_circ_0013561 in NPC, we further carry out research. Firstly, knocking down the hsa_circ_0013561 in HNE1 cells found that it would inhibit cell proliferation, while the expression level of BAX protein was upregulated and the expression level of BCL-2 protein was down-regulated, which promoted cell apoptosis. Change the cell cycle and block cells in G1 phase inhibit migration ability, high expression of E-cadherin and low expression of N-cadherin inhibit EMT progression. The results show that hsa_circ_0013561 can promote the proliferation and migration of NPC.

## Conclusions

**Figure 7:**
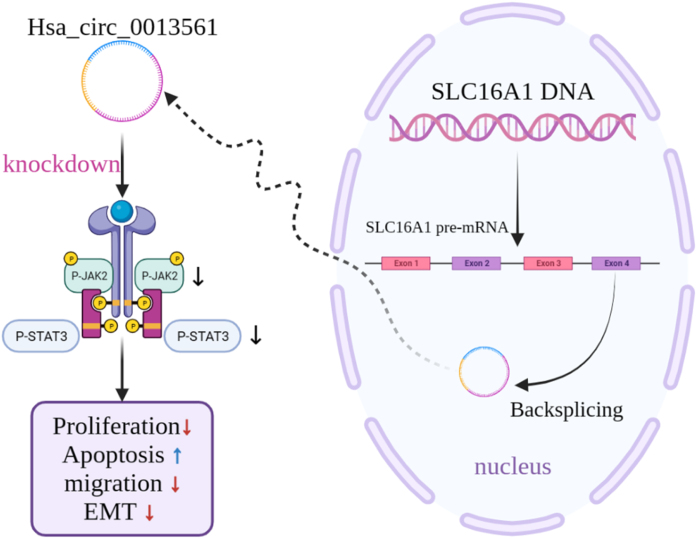
Hsa_circ_0013561 promotes progression of nasopharyngeal carcinoma by activating JAK2/STAT3 signaling pathway.

In conclusion, these data indicate that the expression of hsa_circ_0013561 in NPC is increased and closely related to the malignant progression of NPC. Knocking down hsa_circ_0013561 inhibits NPC progression through the JAK2/STAT3 signaling pathway (Fig. 7). Therefore, our results provide a new target for NPC treatment that deserves further investigation.

## Fundings

This work was supported by the Research Grant for Health Science and Technology of Shanghai Municipal Health Commission [grant number 202140405]; and the Integrated construction of thyroid tumor prevention and treatment in Pudong New Area [grant number PW2019D-4]; and the Key Subspecialty Program of Pudong Health Bureau of Shanghai [grant number PWZy2020-06]; and the Clinical Characteristic Subject of Pudong Health Bureau of Shanghai [grant number PWYts2021-15].

## Conflicts of interest

The authors declare no conflicts of interest.
